# Supplementary material for: Multiplexed functional genomic analysis of 5’ untranslated region mutations across the spectrum of prostate cancer
Source: Nat Commun. 2021 Jul 9;12:4217. doi: 10.1038/s41467-021-24445-6 (PMC8270899; doi:10.1038/s41467-021-24445-6)
Supplement: Supplementary file 1 — Supplementary Information [file 41467_2021_24445_MOESM1_ESM.pdf]

Multiplexed functional genomic analysis of 5' untranslated region mutations across the spectrum of prostate cancer

Yiting Lim[1], Sonali Arora[1], Samantha L. Schuster[1,2], Lukas Corey[1], Matthew Fitzgibbon[3], Cynthia L. Wladyka[1], Xiaoying Wu[4], Ilsa M. Coleman[1], Jeffrey J. Delrow[3], Eva Corey[5], Lawrence D. True[6], Peter S. Nelson[1,7], Gavin Ha[8], Andrew C. Hsieh[1,7]*

[1]Divisions of Human Biology and Clinical Research, Fred Hutchinson Cancer Research Center, Seattle, WA 98109, USA.
[2]Molecular and Cellular Biology Graduate Program, University of Washington, Seattle, WA 98915, USA.
[3]Genomics & Bioinformatics Shared Resource, Fred Hutchinson Cancer Research Center, Seattle, WA 98109, USA.
[4]Division of Basic Sciences, Fred Hutchinson Cancer Research Center, Seattle, WA 98109, USA.
[5]Department of Urology, University of Washington, Seattle, WA 98915, USA.
[6]Department of Pathology, University of Washington, Seattle, WA 98915, USA.
[7]Departments of Medicine and Genome Sciences, University of Washington, Seattle, WA 98195, USA.
[8]Divison of Public Health Sciences, Fred Hutchinson Cancer Research Center, Seattle, WA 98109, USA.

Description of supplementary information

Supplementary information includes 12 Figures.

- Supplementary Figure 1: Lengths of all 326 5' UTRs with somatic mutations in LuCaP PDX samples.
- Supplementary Figure 2: Gland enriched normal prostate tissue used for RNAseq and ribosome profiling.
- Supplementary Figure 3: RNAseq and ribosome profiling of mCRPC PDX tissues.
- Supplementary Figure 4: Validation of PLUMAGE using the luciferase reporter assay.
- Supplementary Figure 5: Comparison of mutation rates in 5' UTRs vs protein coding regions in human prostate cancer.
- Supplementary Figure 6: Most frequently mutated 5' UTR regulatory elements in prostate cancer.
- Supplementary Figure 7: Determining 5' UTR TSSs and polysome profiling in PLUMAGE experiments.
- Supplementary Figure 8: Quantification of 30-bp barcodes in PLUMAGE.
- Supplementary Figure 9: Polysome to total RNA measurements of translationally regulated PLUMAGE hits correlate well with polysome to 80S measurements, and functional 5' UTR mutations are not associated with regional DNA structural changes.
- Supplementary Figure 10: FOS and FGF7 5' UTR mutations increase transcript levels independent of mRNA stability and sequence of the randomer barcode.

- Supplementary Figure 11: Different randomer 30-bp barcode used in PLUMAGE does not impact translation efficiency differences and western blot of CKS2 after knockdown.
- Supplementary Figure 12: Patients with MAP kinase pathway gene mutations that significantly alter gene expression by PLUMAGE were more sensitive to Taxotere therapy.

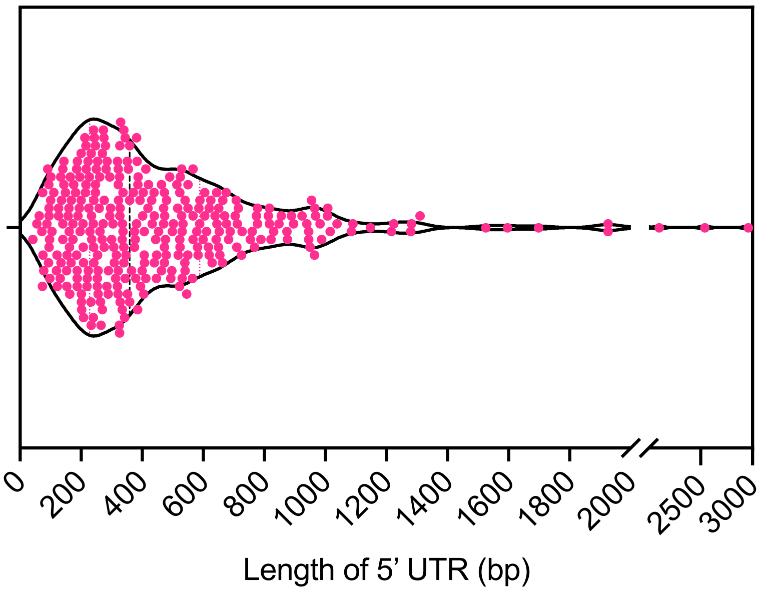

Supplementary Figure 1: Lengths of all 326 5’ UTRs with somatic mutations in LuCaP PDX samples. Each pink dot represents a mutated 5’ UTR. Source data are provided as a Source data file.

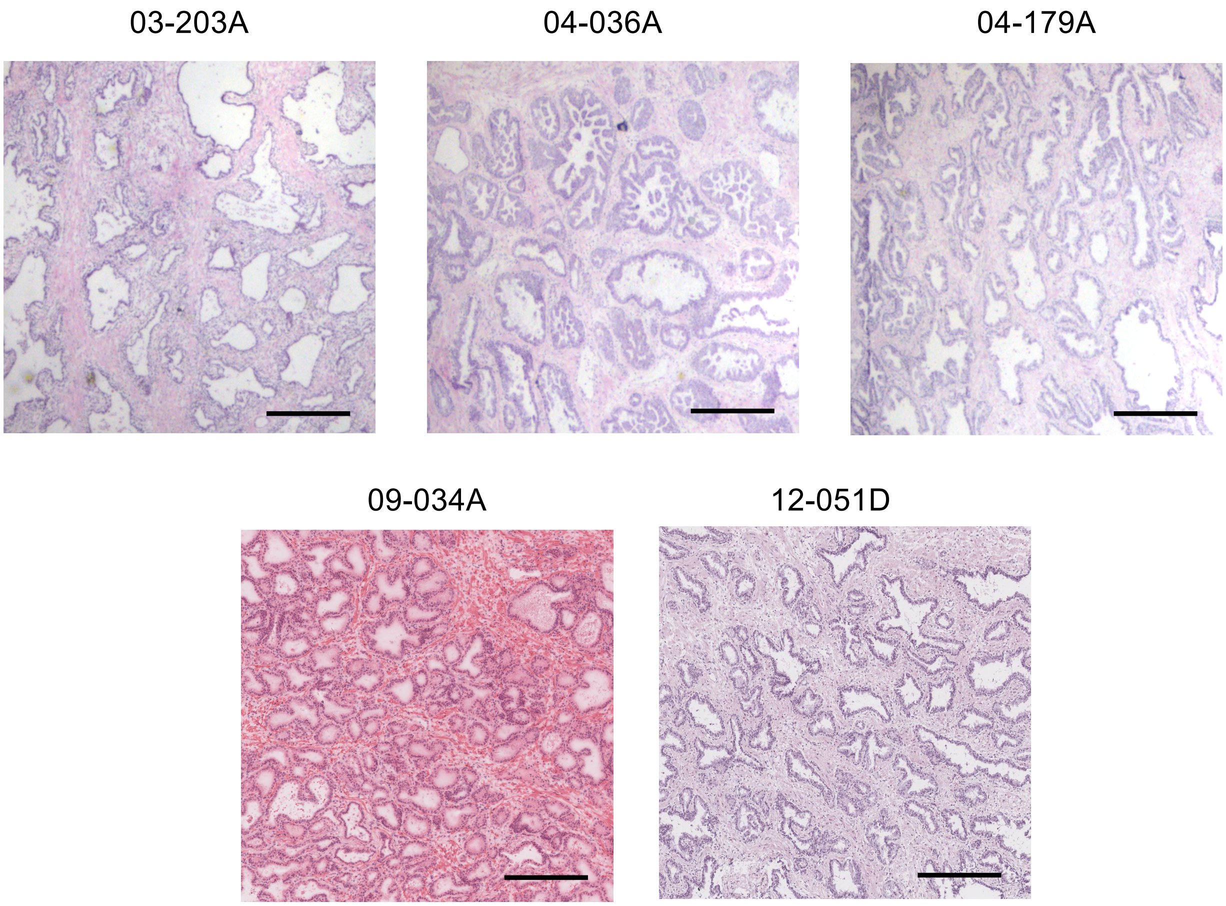

Supplementary Figure 2: Gland enriched normal prostate tissue used for RNAseq and ribosome profiling. Representative hematoxylin and eosin (H&E) staining of normal human prostate tissue from five individuals showing highly glandular tissue that were used in the RNAseq and ribosome profiling experiments in Fig. 1 (Scale bars represent 100 uM).

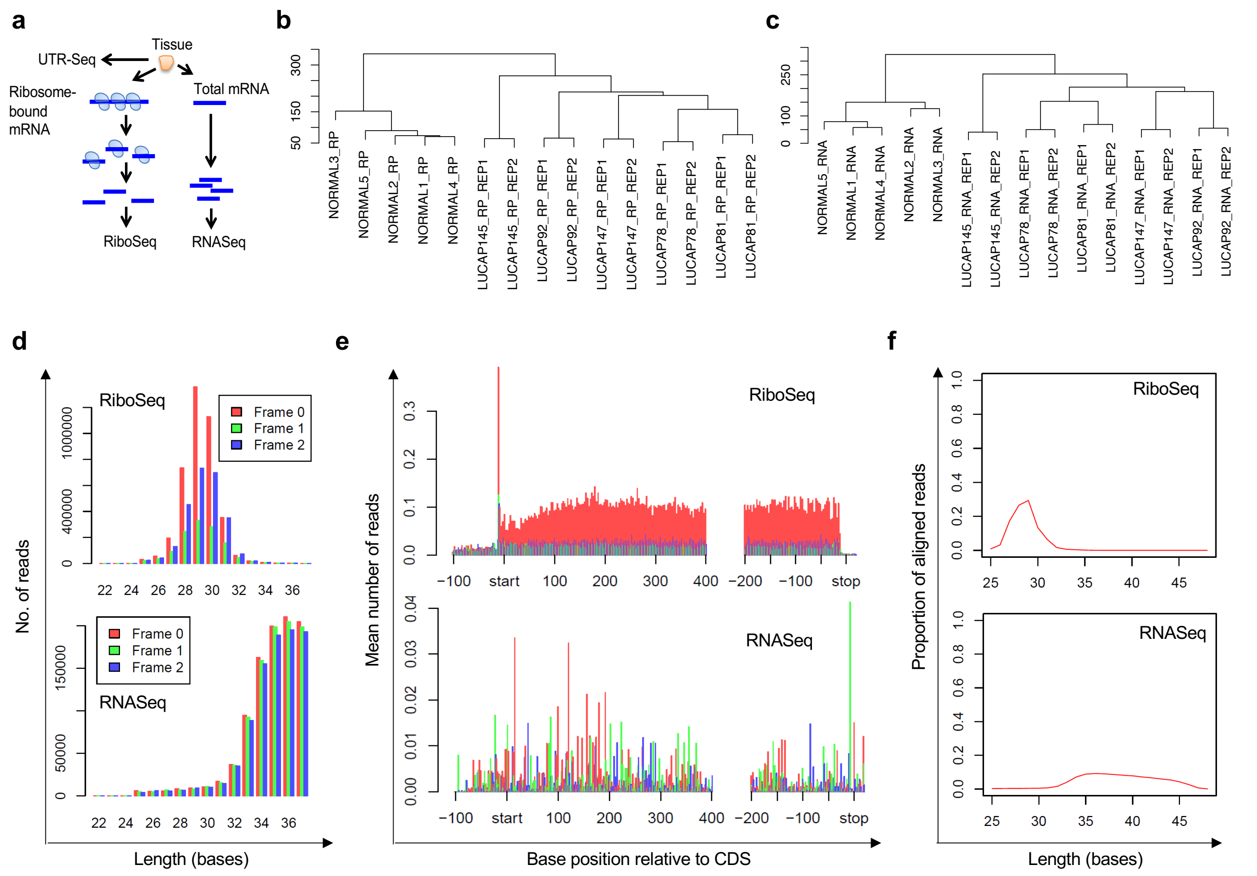

Supplementary Figure 3: RNAseq and ribosome profiling of mCRPC PDX tissues.

(a) Schematic diagram of UTR-Seq and ribosome profiling on the same LuCaP PDX tissue. 5' UTR somatic mutations, RNASeq and ribosome-bound mRNA reads were obtained from each tissue.

(b and c) Dendrograms of normalized read counts for ribosome-bound and total RNA replicates.

(d) Representative periodicity plots of ribosome-bound mRNA and total mRNA from a PDX tissue. To ensure isolated ribosome-bound mRNAs, sequencing libraries were analyzed for triplet periodicity. For each read length specified, the sum of alignments in the different frames is

shown, together with the maximum likelihood frame for ribosome bound (top) and total RNA samples (bottom).

(e) Representative periodicity plots showing ribosome bound fragments enriched in one of the three possible codon frames (top) at each base relative to coding start/end, whereas non-protected total mRNA (bottom) is not.

(f) Representative plots of multiple lengths of sequenced reads for ribosome bound (top) and total RNA samples (bottom). Ribosome footprints around 28-30 bases in length were captured.

Source data are provided as a Source data file.

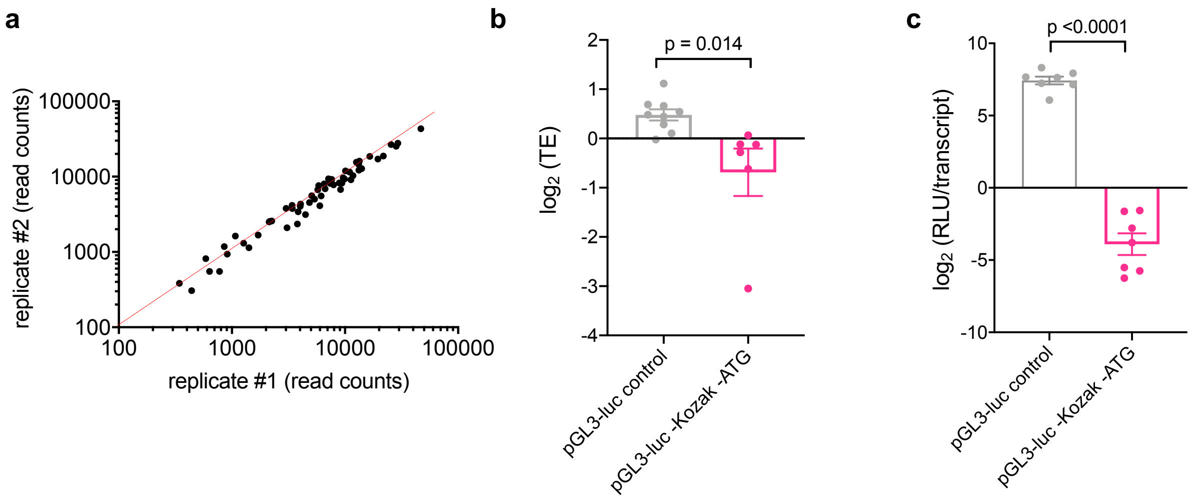

Supplementary Figure 4: Validation of PLUMAGE using a luciferase reporter assay.

(a) Scatter plot showing correlation of normalized read counts per 8-bp barcode between biological replicates in small PLUMAGE library (Pearson r = 0.91, p = 0.0001).

(b) Comparison of performance of a construct without Kozak and ATG sequences (represented in pink) by PLUMAGE. p = 0.014, two-sided Student's t-test (n = 9 biological replicates for pGL3-luc control, n = 6 biological replicates for minus Kozak and minus ATG, data are presented as mean $\pm$ s.e.m.).

(c) Luciferase assay of construct without Kozak and ATG sequences (represented in pink) normalized to the amount of luciferase transcript confirms result seen by PLUMAGE. $p < 0.0001$, two-sided Student's t-test (n = 7 biological replicates, data are presented as mean $\pm$ s.e.m.).

Source data are provided as a Source data file.

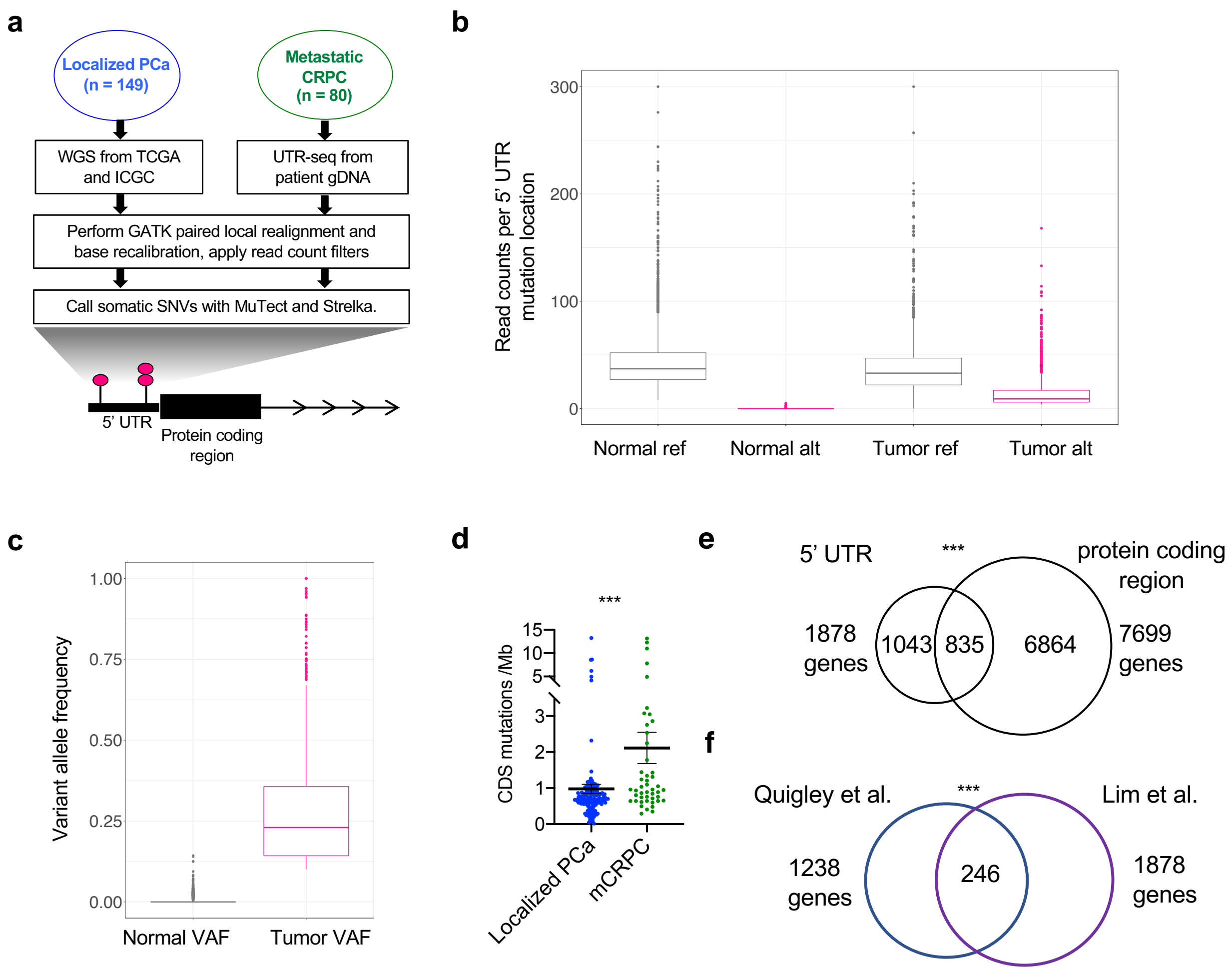

Supplementary Figure 5: Comparison of mutation rates in 5' UTRs vs protein coding regions in human prostate cancer.

(a) Schematic diagram of obtaining 5' UTR somatic mutations from patient samples. Publicly available whole-genome sequencing (WGS) data of localized prostate cancer patients were downloaded and analyzed. Genomic DNA was obtained from mCRPC patients, sequenced, and analyzed.

(b) Number of read counts per reference (ref, represented in grey) and altered (alt, represented in pink) nucleotide in the 5' UTRs of tumor and matched normal samples (n > 2200 mutations).

Tumor alt read counts are much higher than normal alt read counts, suggesting the presence of somatic mutations. Data are presented as median as the center line, and the first and third quartiles as the upper and lower edges of the box.

(c) Variant allele frequency (VAF) of all 5' UTR mutations from tumor (pink) and matched normal (grey) samples show higher tumor VAFs compared to normal tissues, suggesting reliable detection of somatic 5' UTR mutations ($n > 2200$ mutations). Data are presented as median as the center line, and the first and third quartiles as the upper and lower edges of the box.

(d) Comparison of CDS mutation rates between localized prostate cancer ($n = 147$ localized prostate cancer patients, each blue dot represents one patient) and mCRPC patients ($n = 47$ mCRPC patients, each green dot represents one patient) shows higher mutation rates in mCRPC patients ($p = 0.0001$, two-tailed Mann Whitney test). Data are presented as mean values $\pm$ s.e.m.

(e) Number of genes with mutations in 5' UTRs and CDS regions show some overlap. At least 50% of genes have mutations that are exclusive to either the 5' UTR or the CDS (***$p = 0.0001$, Hypergeometric test).

(f) Number of genes with mutations in 5' UTRs in the Quigley et al. Cell 2018[26] dataset compared to our dataset (***$p = 0.0001$, Hypergeometric test).

Source data are provided as a Source data file.

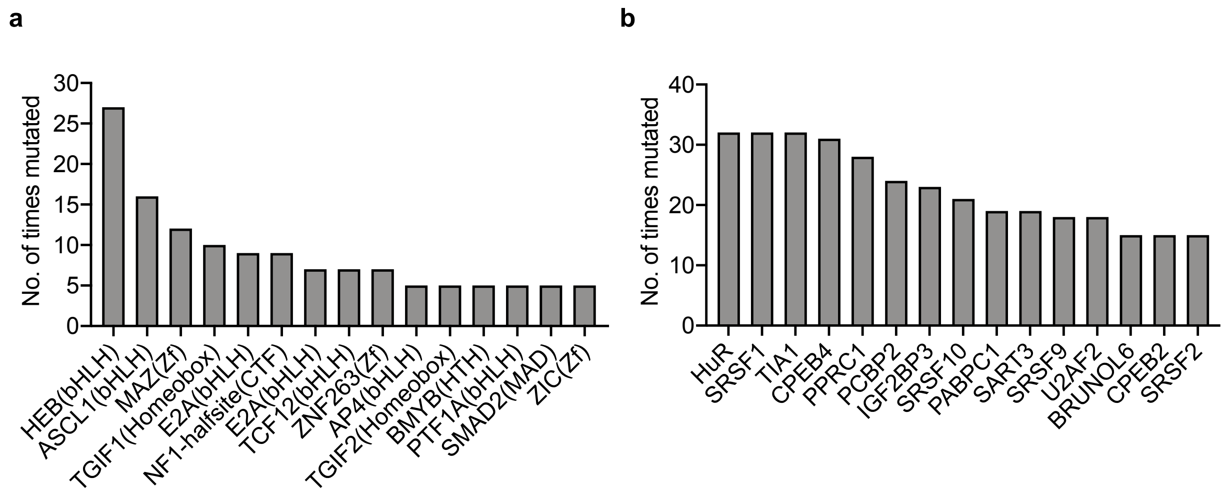

Supplementary Figure 6: Most frequently mutated 5’ UTR regulatory elements in prostate cancer.

(a) Most frequently mutated 5’ UTR DNA binding elements in our prostate cancer patient cohort identified using the HOMER database.

(b) Most frequently mutated RNA binding protein elements in our prostate cancer patient cohort identified using the Hughes database.

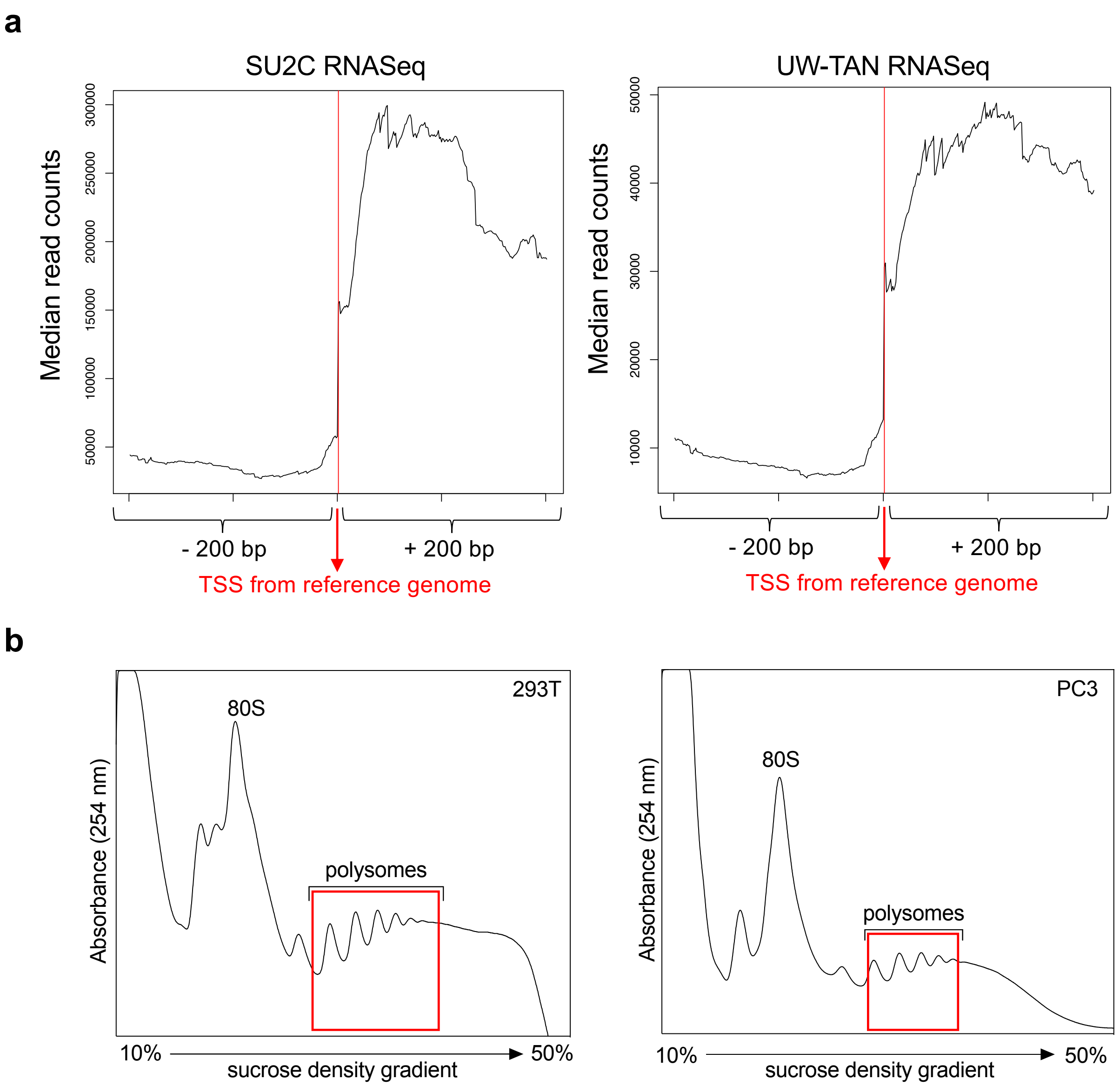

Supplementary Figure 7: Determining 5' UTR TSSs and polysome profiling in PLUMAGE experiments.

(a) Read counts from RNASeq of mCRPC patients relative to transcription start sites of genes from reference genome (Refseq) from two separate RNASeq datasets. SU2C RNASeq was obtained from publicly available data from Robinson et al. Cell 2015[1], whereas the UW-TAN RNASeq was obtained from Kumar et al. Nat Med 2016[2] and were from mCRPC patients we

sequenced. All 5' UTRs assayed in PLUMAGE were compared and had high read counts at the TSS (indicated by red arrow) suggesting robust expression in prostate cancer tissue.

(b) Representative polysome profiling traces from 293T cells and PC3 cells transfected with PLUMAGE plasmid library. The polysome fractions after the disome were pooled (indicated in red box shown in figure) to obtain polysome-bound mRNA for each replicate.

Source data are provided as a Source data file.

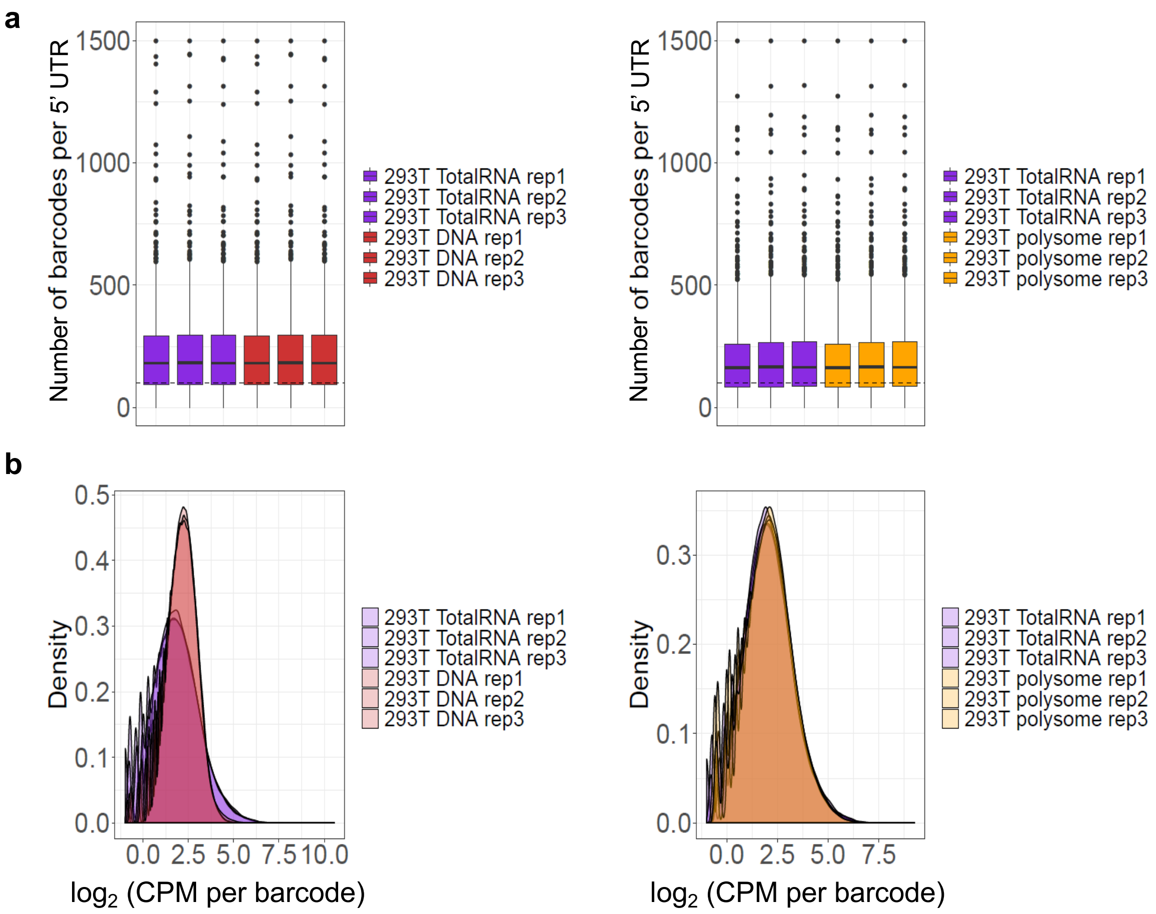

Supplementary Figure 8: Quantification of 30-bp barcodes in PLUMAGE.

(a) Number of unique 30-bp barcodes per mutated and unmutated 5’ UTR sequence in each sample, for each quantitative measurement as determined by taking ratio of total mRNA/DNA and polysome/total mRNA. Data are presented as median as the center line, and the first and third quartiles as the upper and lower edges of the box.

(b) Density plots of normalized read counts per barcode, for each quantitative measurement determined by taking ratio of total mRNA/DNA and polysome/total mRNA. Data shown for all three biological replicates and is representative of both cell lines.

Source data are provided as a Source data file and Supplementary Data 6c.

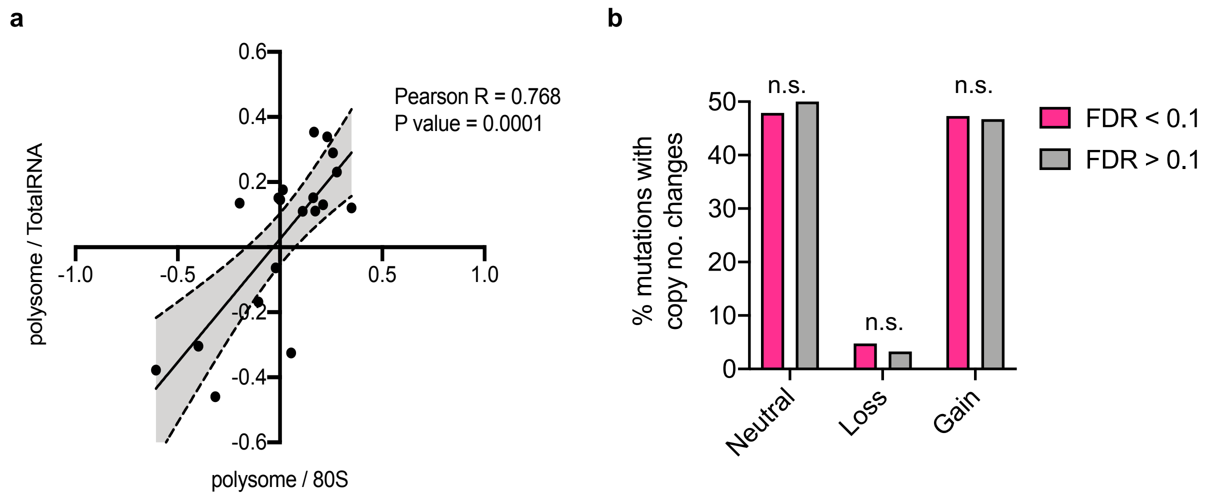

Supplementary Figure 9: Polysome to total RNA measurements of translationally regulated PLUMAGE hits correlate well with polysome to 80S measurements, and functional 5' UTR mutations are not associated with regional DNA structural changes.

(a) Ratio of polysome-bound mRNA read counts to total mRNA read counts correlates well with ratio of polysome-bound mRNA read counts to 80S-bound read counts in PLUMAGE. Each dot represents a 5' UTR mutation found to have significant change in translation efficiency by PLUMAGE (FDR < 0.1). Pearson correlation coefficient was calculated, R = 0.768, p value = 0.001.

(b) Copy number analysis of the regional 5' UTR genomic structure from patients with functional (FDR < 0.01, pink) and non-functional (FDR > 0.01, grey) mutations at determined by PLUMAGE. n.s. = not statistically significant, two-sided Chi-square test.

Source data are provided as a Source data file.

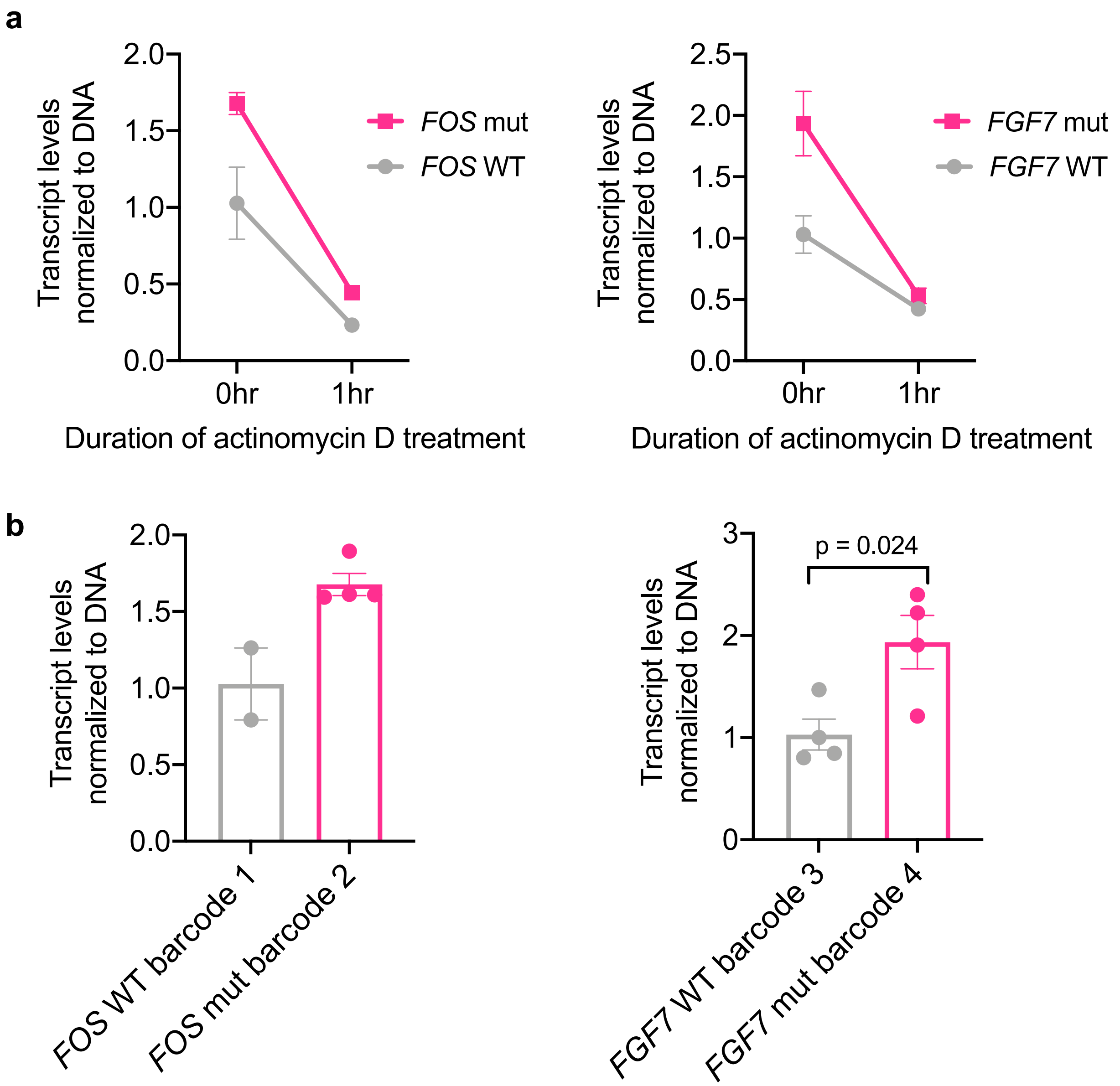

Supplementary Figure 10: FOS and FGF7 5' UTR mutations increase transcript levels independent of mRNA stability and sequence of the randomer barcode.

(a) Amount of transcript at 0 hr and 1 hr of 10 μM actinomycin D treatment 48 hours after transfection of *FOS* and *FGF7* WT (represented in grey) and mutant (represented in pink) plasmids. Rate of mRNA degradation between WT and mutant samples do not impact the

increase in transcript levels brought about by the mutations (n = 2 biological replicates for *FOS*, n = 3-4 biological replicates for *FGF7*, data are presented as mean $\pm$ s.e.m).

(b) *FGF7* and *FOS* 5' UTR mutations (represented in pink) show increase in transcript levels by qPCR, even with different 30-bp barcodes attached to the 3' end of the luciferase gene. One-sided Student's t-test (n = 2 biological replicates for *FOS* WT, n = 4 biological replicates for *FOS* mutant, *FGF7* WT and mutant, data are presented as mean $\pm$ s.e.m).

Source data are provided as a Source data file.

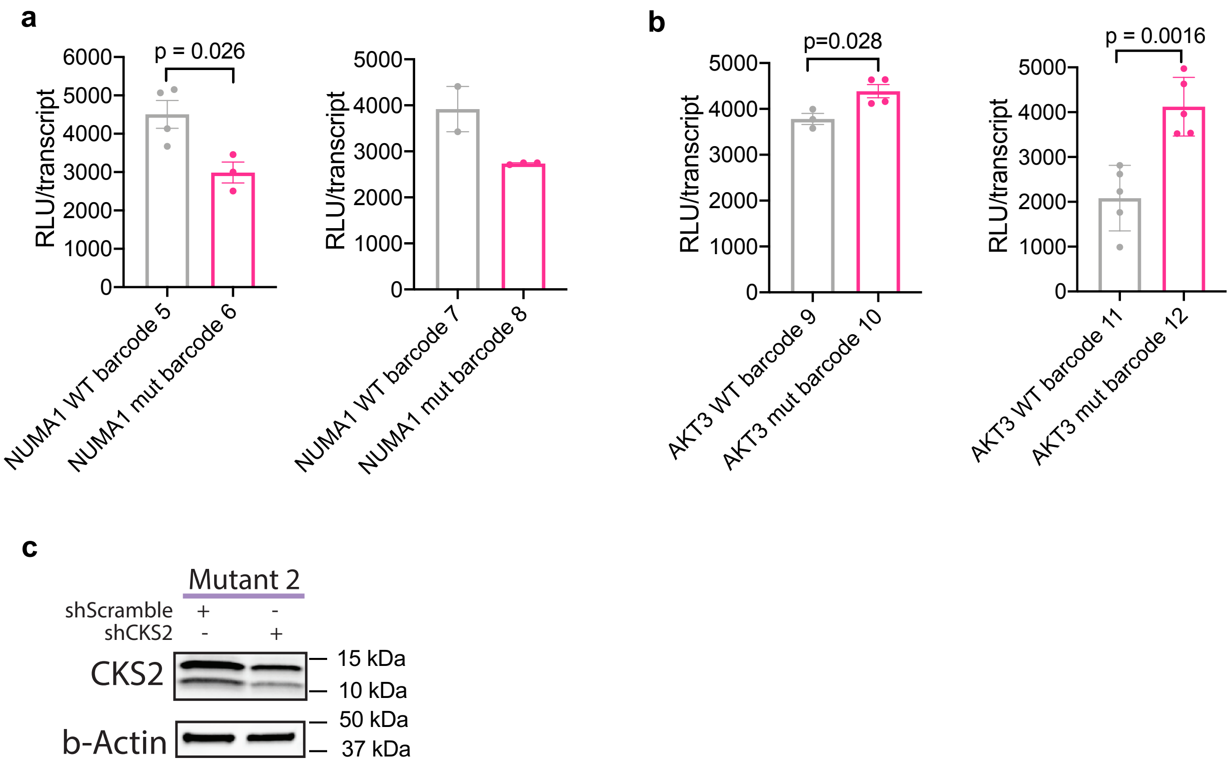

Supplementary Figure 11: Different randomer 30-bp barcode used in PLUMAGE does not impact translation efficiency differences and western blot of CKS2 after knockdown.

(a) Different 30-bp barcodes in *NUMA1* WT (represented in grey) and mutant (represented in pink) plasmids do not affect the decrease in translation efficiency as a result of the mutation (C -> A, chr11: 71780891). Data shows 4 different barcodes (barcodes 5, 6, 7, 8) performed in biological replicates (n = 4 for barcode 5, n = 3 for barcode 6, n = 2 for barcode 7, n = 3 for barcode 8, data are presented as mean $\pm$ s.e.m). P values were calculated using the one-sided Student's t test.

(b) Different 30-bp barcodes in *AKT3* WT (represented in grey) and mutant (represented in pink) plasmids do not affect the increase in translation efficiency as a result of the mutation (C -> T,

chr1: 244006547). Data shows 4 different barcodes (barcodes 9, 10, 11, 12), performed in biological replicates (n = 3 for barcode 9, n = 4 for barcode 10, n = 5 for barcode 11, n = 5 for barcode 12, data are presented mean $\pm$ s.e.m). P values were calculated using the one-sided Student's t test.

(c) Immunoblot of CKS2 5' UTR knock-in mutant cell line after shRNA knockdown of CKS2 demonstrates the specificity of the antibody. Data is representative of 2 independent experiments with similar results.

Source data are provided as a Source data file.

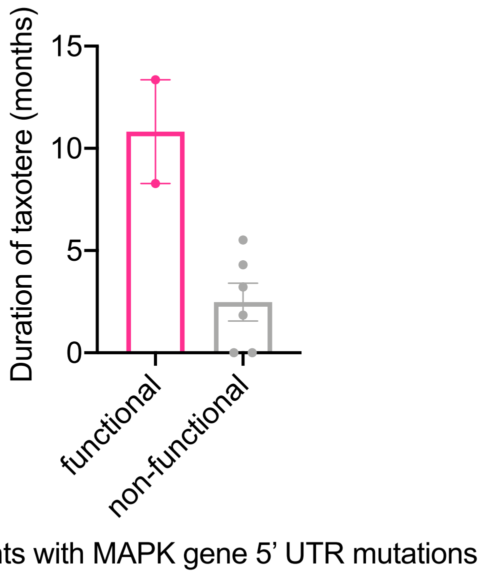

Supplementary Figure 12: Patients with MAP kinase pathway gene mutations that significantly alter gene expression by PLUMAGE were more sensitive to Taxotere therapy. Comparison of patients with functional MAP kinase regulator hits by PLUMAGE (FDR < 0.1) (n = 2 patients, represented in pink) versus patients with non-functional MAP kinase pathway 5' UTR mutations (FDR > 0.1) (n = 6 patients, represented in grey, data are presented as mean ± s.e.m.). Source data are provided as a Source data file.

Supplementary References

1. Robinson, D. et al. Integrative clinical genomics of advanced prostate cancer. *Cell* **161**, 1215 – 1228 (2015).
2. Kumar, A. et al. Substantial interindividual and limited intraindividual genomic diversity among tumors from men with metastatic prostate cancer. *Nat. Med.* **22**, 369 – 378 (2016).
