# Supplementary material for: Multiplexed functional genomic analysis of 5’ untranslated region mutations across the spectrum of prostate cancer
Source: Nat Commun. 2021 Jul 9;12:4217. doi: 10.1038/s41467-021-24445-6 (PMC8270899; doi:10.1038/s41467-021-24445-6)
Supplement: Supplementary file 2 — Descriptions of Additional Supplementary Files [file 41467_2021_24445_MOESM2_ESM.pdf]

Descriptions of Additional Supplementary Files

**Supplementary Data 1**
**Description:** Ribosome profiling analysis of five LuCaP PDXs. a) Somatic 5' UTR mutations of 5 LuCaP models. b) RNAseq analysis of LuCaP models compared to 5 normal human controls. c) Translation efficiency analysis of LuCaP models compared to 5 normal human controls.

**Supplementary Data 2**
**Description:** Sequences of 8-bp barcodes used in small proofof-concept PLUMAGE library.

**Supplementary Data 3**
**Description:** Prostate cancer patient information and depth of sequencing per patient. Sequencing and mapping metrics for patient specimens from a) Fred Hutch/UW, b) TCGA, c) ICGC. ·

**Supplementary Data 4**
**Description:** UTR sequencing details and mutated gene lists. a) Number of nucleotide specific 5' UTR mutations across 229 patients. b) Number of nucleotide specific coding sequence (CDS) mutations across 229 patients. c) Somatic 5' UTR mutations across our patient cohort. d) Somatic CDS mutations across our patient cohort. e) Genes with 5' UTR mutations across all samples, localized samples, and metastatic samples. f) Genes with CDS mutations in all samples. g) List of recurrent 5' UTR mutations.

**Supplementary Data 5**
**Description:** DNA and RNA binding element motif sequences. a) Number of times 5' UTR mutations impact DNA binding elements. b) Number of times 5' UTR mutations impact RNA binding protein motifs.

**Supplementary Data 6**
**Description:** PLUMAGE analysis and sequences. a) 5' UTR sequences tested in PLUMAGE. b) Overview of short read sequencing counts for large PLUMAGE analysis. c) Raw data for histogram plots in Supplementary Figure 8b. d) Transcript-specific changes observed by PLUMAGE. e) Translation efficiency changes observed by PLUMAGE.

**Supplementary Data 7**
**Description:** RNA binding element motif sequences from CISBP database.

**Supplementary Data 8**
**Description:** Primer sequences used in PLUMAGE short read sequencing and all other primers.
