# Supplementary material for: Multiplexed functional genomic analysis of 5’ untranslated region mutations across the spectrum of prostate cancer
Source: Nat Commun. 2021 Jul 9;12:4217. doi: 10.1038/s41467-021-24445-6 (PMC8270899; doi:10.1038/s41467-021-24445-6)
Supplement: Supplementary file 12 — Reporting Summary [file 41467_2021_24445_MOESM12_ESM.pdf]

nature research

Corresponding author(s): Andrew C. Hsieh

Last updated by author(s): May 24, 2021

Reporting Summary

Nature Research wishes to improve the reproducibility of the work that we publish. This form provides structure for consistency and transparency in reporting. For further information on Nature Research policies, see our Editorial Policies and the Editorial Policy Checklist.

Statistics

For all statistical analyses, confirm that the following items are present in the figure legend, table legend, main text, or Methods section.

n/a	Confirmed	
☐	☒	The exact sample size (*n*) for each experimental group/condition, given as a discrete number and unit of measurement
☐	☒	A statement on whether measurements were taken from distinct samples or whether the same sample was measured repeatedly
☐	☒	The statistical test(s) used AND whether they are one- or two-sided<br>*Only common tests should be described solely by name; describe more complex techniques in the Methods section.*
☐	☒	A description of all covariates tested
☐	☒	A description of any assumptions or corrections, such as tests of normality and adjustment for multiple comparisons
☐	☒	A full description of the statistical parameters including central tendency (e.g. means) or other basic estimates (e.g. regression coefficient) AND variation (e.g. standard deviation) or associated estimates of uncertainty (e.g. confidence intervals)
☐	☒	For null hypothesis testing, the test statistic (e.g. *F*, *t*, *r*) with confidence intervals, effect sizes, degrees of freedom and *P* value noted<br>*Give P values as exact values whenever suitable.*
☒	☐	For Bayesian analysis, information on the choice of priors and Markov chain Monte Carlo settings
☒	☐	For hierarchical and complex designs, identification of the appropriate level for tests and full reporting of outcomes
☐	☒	Estimates of effect sizes (e.g. Cohen's *d*, Pearson's *r*), indicating how they were calculated

Our web collection on statistics for biologists contains articles on many of the points above.

Software and code

Policy information about availability of computer code

Data collection	TopHat (v2.0.14), Bowtie (v1.0.0) were used for alignment. Counting reads for gene associations against the UCSC genes was done with HTSeq (v 0.11.0). ContEst (v3.6) was used to determine patient sample cross contamination.<br>Xtail (v1.1.5) and DESeq2 (v1.26.0) were both used to find translationally and transcriptionally regulated genes.<br>R version (3.3.2) was used for all analysis. The following R packages were used: edgeR (v3.28.1), ggplot2 (v 2.2.1), pheatmap (v1.0.12). Mutect (v1) was used to call SNV mutations. Strelka (v1) was used to call SNV and small indel mutations. Sequenza (v 2.1.9999b) was used to estimate allele-specific copy number calls. MSigDB (v 1.7) was used to compute overlaps with KEGG gene sets. PacBio Sequel v3.0 was used for long-read sequencing.
Data analysis	Xtail (v1.1.5) was used to find translationally regulated genes individually for each LuCaP (FDR < 0.1 and fold change > 1.5). DESeq2 (v 1.26.0) was used to find transcriptionally regulated genes individually for each LuCaP (FDR < 0.05 and fold change > 2), which were excluded from the translationally regulated gene lists. R/Bioconductor package, riboseqR (v1.26.0) was used to calculate triplet periodicity in all samples.<br>Mutect (v1) and Strelka (v1) were used to call mutations, annovar was used to annotate mutations. Cytoscape (v3.7.2) was used for visualizing gene networks. All mutation and ribosome profiling analysis was done in R and scripts can be found at: https://github.com/sonali-bioc/Lim-5utr-Paper Python scripts for functional impacts of translation and transcription are available at : https://github.com/lukascorey/5-UTR-Mutation-Analysis

For manuscripts utilizing custom algorithms or software that are central to the research but not yet described in published literature, software must be made available to editors and reviewers. We strongly encourage code deposition in a community repository (e.g. GitHub). See the Nature Research guidelines for submitting code & software for further information.

Data

Policy information about availability of data

All manuscripts must include a data availability statement. This statement should provide the following information, where applicable:

- Accession codes, unique identifiers, or web links for publicly available datasets
- A list of figures that have associated raw data
- A description of any restrictions on data availability

All data needed to evaluate the conclusions in the paper are described in the paper and/or the methods, and are available at the following repositories. Human mCRPC UTR sequencing data (Figure 2a, c, d) were deposited in dbGaP under accession phs001825.v1.p1 (https://www.ncbi.nlm.nih.gov/projects/gap/cgi-bin/study.cgi?study_id=phs001825.v1.p1). RNASeq, Ribosome profiling (Figures 1b, c), Exome-seq data (Figure 1a) for Patient-derived xenografts, and PLUMAGE short-read sequencing data (Figure 3b, c, d, e) can be accessed at Gene Expression Omnibus (GEO) under SuperSeries GSE149489 (https://www.ncbi.nlm.nih.gov/geo/query/acc.cgi?acc=GSE149489). Source data are available with this paper as a Source data file. The remaining data are available within the Article, Supplementary Information or available from the authors upon request.

Field-specific reporting

Please select the one below that is the best fit for your research. If you are not sure, read the appropriate sections before making your selection.

☒ Life sciences ☐ Behavioural & social sciences ☐ Ecological, evolutionary & environmental sciences

For a reference copy of the document with all sections, see nature.com/documents/nr-reporting-summary-flat.pdf

Life sciences study design

All studies must disclose on these points even when the disclosure is negative.

Sample size	For UTR sequencing, sample sizes were limited to what whole-genome sequencing data were publicly available for localized prostate cancer patients, and how many mCRPC samples were available from the University of Washington at the time of our study. For the PLUMAGE library, the goal was to test as many mutations deemed to be functional, which was why we included all recurrently mutated 5' UTRs found in 2 or more patients, and cancer-associated genes. For all validation experiments, including the CRISPR experiments, at least 3 biological replicates were performed to obtain a p-value using the Student's t-test. For data involving mCRPC patients with MAPK mutations, we were limited to the number of patients who harbored those mutations, as well as the availability of RNA sequencing data for each patient.
Data exclusions	For the PLUMAGE library long-read sequencing analysis, within each circular consensus sequence, we identified the 5' UTR and associated 30-bp barcode by searching for flanking 20-bp sequences expected to be constant across all constructs. CCS sequences where these flanking sequences were not found, or where a barcode had not been inserted and the EcoRI target sequence GAATTC remained, were excluded from further consideration. 117,874 barcodes that did not match an expected 5’ UTR were excluded from further analysis. Of these, 50% were supported by a single circular consensus sequence only so that multiple independent circular consensus sequences were unavailable for multiple alignment and further refinement.
Replication	For ribosome profiling experiments, each sample was performed in duplicates. For PLUMAGE, three biological replicates were performed for each cell line. For validation experiments with individual 5'UTRs in luciferase reporter constructs, at least 3 to 6 biological replicates were performed. For CRISPR base-editing experiments, 3 replicates were performed. All attempts at replication were successful. All other experiments not mentioned here were performed independently at least 3 times, and all attempts at replication were successful.
Randomization	Most of the samples in our study were not randomly allocated into experimental groups, and had no covariates. For instance, in ribosome profiling experiments, tumors were compared to normal prostate tissue. For PLUMAGE and validation experiments of individual 5' UTRs, the unmutated 5'UTR was compared to its mutated counterpart. Each 5' UTR length (unmutated and mutated) were kept constant. The 30-bp barcodes in the large PLUMAGE library were generated randomly by cloning. For mCRPC patients harboring specific MAP kinase 5'UTR mutations vs patients who don't have those specific mutations, the mutation rate of these patients were taken into consideration. Besides these experimental considerations, all other samples were not randomly allocated. Furthermore, our study had no active participants that had to be allocated into experimental groups. The patient samples we sequenced (tumor and matched normal) were from post-mortem samples. For the cis-element mutation analysis, the background distribution was generated by performing ~10,000 permutations of all 5’ UTR mutation locations found within our dataset. The original mutational frequency of all specific transversions, transitions and trinucleotide context (a total of 288 possible mutations are possible under this scheme—64 possible codons plus 32 additional with no nucleotide in exclusively the first or third position, each with three possible mutations to the middle base) were taken into account as covariates.
Blinding	Blinding was not possible for this study. Localized vs metastatic prostate cancer patient samples had to be identified and compared in separate groups.

Reporting for specific materials, systems and methods

We require information from authors about some types of materials, experimental systems and methods used in many studies. Here, indicate whether each material, system or method listed is relevant to your study. If you are not sure if a list item applies to your research, read the appropriate section before selecting a response.

Materials & experimental systems

n/a	Involved in the study
☐	☒ Antibodies
☐	☒ Eukaryotic cell lines
☒	☐ Palaeontology and archaeology
☒	☐ Animals and other organisms
☐	☒ Human research participants
☒	☐ Clinical data
☒	☐ Dual use research of concern

Methods

n/a	Involved in the study
☒	☐ ChIP-seq
☒	☐ Flow cytometry
☒	☐ MRI-based neuroimaging

Antibodies

Antibodies used	CKS2 (Abcam ab155078, 1:1000 dilution) and beta-actin (Sigma-Aldrich A5316, 1:1000 dilution)
Validation	We used a 1:1000 dilution for the CKS2 antibody, as recommended by the manufacturer, and observed a band at the correct size. To validate this, we performed shRNA knockdown of CKS2 in 293T cells, and showed a decrease in CKS2 protein expression in at least 2 independent experiments. Uncropped western blot images can be found in the Source data file. For the beta-actin monoclonal antibody, we followed the manufacturer's instructions, and our western blots showed a distinct band at the right size as determined by the manufacturer.

Eukaryotic cell lines

Policy information about cell lines

Cell line source(s)	HEK 293T, PC3 (both from ATCC)
Authentication	Both cell lines were authenticated by short tandem repeat profiling and matched to STR profiles from the ATCC database for human cell lines.
Mycoplasma contamination	Both cell lines tested negative for the presence of mycoplasma.
Commonly misidentified lines (See ICLAC register)	No commonly misidentified cell lines were used.

Human research participants

Policy information about studies involving human research participants

Population characteristics	Samples were obtained postmortem from patients who died of metastatic castration resistant prostate cancer (mCRPC). These patients were all elderly male, age 60 and above, who had metastatic castration resistant prostate cancer, and subjected to a range of different therapies, with metastases in different organ sites.
Recruitment	All mCRPC patients in the study signed written informed consent for a rapid autopsy performed within 6 hours of their death, under the aegis of the Prostate Cancer Donor Program at the University of Washington. The inclusion criteria for the Prostate Cancer Donor Program included a diagnosis of prostate cancer, informed consent to perform an autopsy and informed consent to use biospecimens for biomedical research. The participants are all self-selected, since they had to consent to be enrolled in this study, therefore there could exist self-selection biases. However, the samples from the participants are obtained postmortem, and the analysis can be done in an unbiased way, therefore the self-selection aspect may not impact the results.
Ethics oversight	The Institutional Review Board (IRB) of the University of Washington and the Fred Hutchinson Cancer Research Center approved all procedures involving human subjects.

Note that full information on the approval of the study protocol must also be provided in the manuscript.
